# Robotic-assistance is associated with better joint outcomes compared to conventional techniques in surgically routine total hip arthroplasty: a propensity-matched large database study of 3948 patients

**DOI:** 10.1007/s00402-024-05628-4

**Published:** 2025-01-07

**Authors:** Aakash K. Shah, Monish S. Lavu, Robert J. Burkhart, Christian J. Hecht, Collin Blackburn, Nicholas Romeo

**Affiliations:** 1 Case Western Reserve University School of Medicine, Cleveland, OH 44106 USA; 2 Department of Orthopaedic Surgery, University Hospitals, Cleveland, OH 44106 USA; 3 Department of Orthopaedic Surgery, MetroHealth Medical Center, Cleveland, OH 44106 USA

**Keywords:** Total hip arthroplasty, Robotic-assisted total hip arthroplasty, Conventional total hip arthroplasty, Adverse events, Complications

## Abstract

**Introduction:**

The outcomes of total hip arthroplasty (THA) are highly dependent upon the restoration of native hip biomechanics and optimal component positioning. Robotic technologies for THA have rapidly improved the accuracy of component positioning and maintaining the planned center of rotation. While robotic-assisted THA (RA-THA) has primarily been employed in surgically intricate cases, its potential benefits in scenarios of diminished surgical complexity remain less explored. Therefore, the purpose of this study was to assess the odds of developing systemic and joint complications following RA-THA in cases of reduced surgical complexity.

**Methods:**

A retrospective cohort study was conducted using the TriNetX national database to identify patients who underwent primary THA (Current Procedural Terminology code 27,130) and more specifically RA-THA identified by ICD-10-PCS code 8E0Y0CZ and Healthcare Common Procedure Coding System code S2900 from 2013 to 2022. One-to-one propensity score matching was conducted to generate 2 cohorts: (1) RA-THA and (2) conventional THA (C-THA). Systemic and joint complications were assessed at the 30-day, 90-day, 1-year, and 5-year postoperative periods.

**Results:**

Patients undergoing RA-THA had a lower risk of needing a revision THA at the 90-day, 1-year, and 5-year time points. RA-THA was associated with a lower risk of prosthetic dislocation at 90 days and 1 year and prosthetic pain at 1 year and 5 years. Dislocation of the hip or fracture of the femur was significantly lower in the RA-THA cohort at all four-time points. Patients undergoing RA-THA had a lower risk of developing deep vein thrombosis at 5 years.

**Conclusion:**

These findings suggest that RA-THA has comparable systemic and less joint complication risks at 30-day to 5-year timepoints between RA-THA and C-THA. Future studies with large sample sizes and long-term follow-up are needed to understand the patient-reported outcomes and functional outcomes of RA-THA for cases with reduced surgical complexity.

## Introduction

Total hip arthroplasty (THA) is a commonly performed orthopaedic procedure with the estimated annual volume increasing by 177% between 2000 and 2019 and expected to increase by another 176% by 2040 [1, 2]. One pillar to predict long-term outcomes of THA is the restoration of native hip biomechanics which is based on optimal component positioning. Increased wear, poor function, and joint instability are associated with poor component positioning [3–7]. The latest developments in THA have revolved around technologies to better optimize component positioning and preserve the natural kinematics of the hip [8].

To improve surgical accuracy, precision, and ultimately survivorship, robotic-assisted THA (RA-THA) systems have been developed. RA-THA utilizes patient-specific anatomical data with recommendations for bone resection and ideal component positioning [9]. It is estimated that RA-THA is currently utilized in 5.1% percent of THA cases and continues to grow at a significant rate [10, 11]. Several studies have found these systems to improve acetabular cup positioning [11–13], maintain the planned center of rotation [14–16], reduce the rate of revision for dislocation [17], and decrease postoperative patient costs by thousands of dollars [17–19]. Some of the reported challenges associated with the use of robotic systems are the reported learning curve ranging from 10 to 35 procedures and associated start-up costs [20, 21]. Prior studies do not report the postoperative complications following a RA-THA for surgically routine cases and fail to match cohorts when comparing outcomes between RA-THA and conventional THA (C-THA). Propensity-matching a large cohort of routine RA-THA cases may enable a better comparison to evaluate potential adverse events associated with routine utilization of RA-THA. Furthermore, this will showcase the true advantage of RA-THA in reducing surgical complications during routine THA for the first time.

RA-THA is known to have a longer operative time and optimize the natural joint kinematics which can potentially increase postoperative VTE rates, risk of infection and decrease postoperative revision rates. Therefore, the purpose of this study was to assess the odds of developing medical and orthopaedic adverse events up to a 5-year postoperative period in patients undergoing RA-THA in routine THA cases.

## Methods

### Data source

The US collaborative network of the TriNetX platform (Cambridge, Massachusetts), a federated health research network including electronic health record (EHR) data on patients from 57 healthcare systems within the United States, was utilized. This database contains de-identified data that is continuously compiled from each participating healthcare organization’s EHR including patient demographics, diagnoses, procedures, and medications[22, 23]. All data utilized in this study was de-identified, and therefore, did not require Institutional Review Board (IRB) approval. Additionally, studies utilizing the TriNetX database were determined to be exempt from Western Institutional Review Board (IRB) approval by a qualified expert as defined in Section §164.514(b)(1) of the HIPAA Privacy Rule.

### Patient selection

**Fig. 1:**
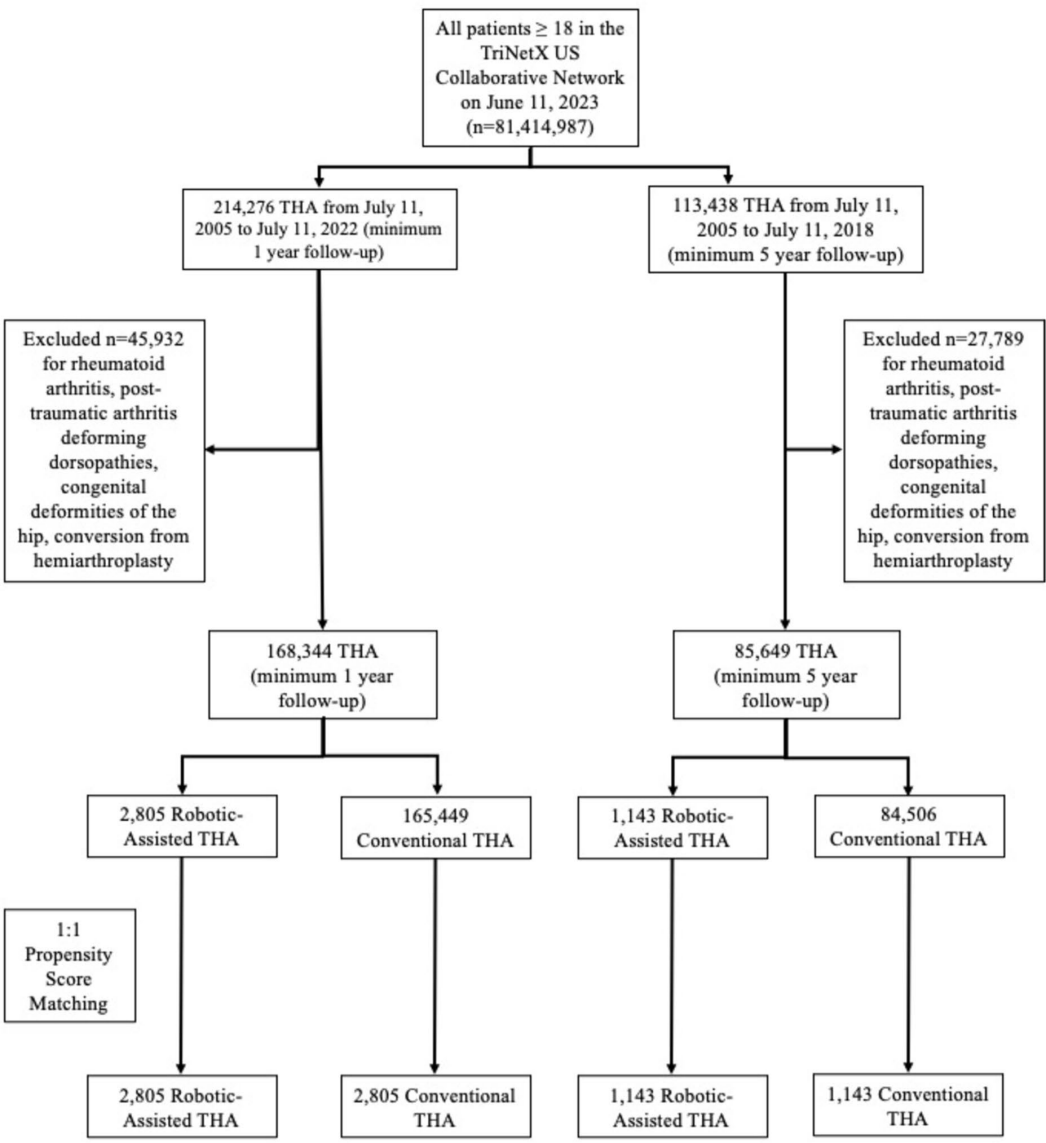
STROBE diagram depicting the patient selection process. *THA* total hip arthroplasty

On July 11th, 2023, International Classification of Diseases, Tenth Revision, Procedural Coding System (ICD-10-PCS) 0SR9 and 0SRB as well as Current Procedural Terminology (CPT) 27,130 codes for THA were used to identify 214,276 patients who received THA between July 11th, 2005, and July 11th, 2022, amongst 81,414,987 total patients in the US collaborative network who were at least 18 years old. These patients had at least 1 year of follow-up. We excluded patients with a diagnosis of rheumatoid arthritis, post-traumatic arthritis, deforming dorsopathies, congenital deformities of the hip, and conversion from hemiarthroplasty to remove all potentially surgically complex cases due to the patient’s anatomy and joint mechanics. After applying these exclusion criteria, 168,344 THA patients undergoing surgically routine THA remained for analysis. Healthcare common procedural coding system (HCPCS) code S2900 and ICD-10-PCS code 8E0Y0CZ were used to separate the 2,805 patients who received robotic-assisted THA and the 165,449 patients who received conventional THA. Following 1:1 propensity score-matching, there were 2,805 patients in both cohorts for analysis (Fig. 1).

For a 5-year subanalysis, 113,438 patients above the age of 18 who received THA between July 11, 2005, and July 11, 2018, were identified. These patients had at least 5 years of follow-up. After excluding 27,789 patients with the same aforementioned exclusion criteria, 85,649 surgically routine THA patients remained for analysis. Of whom, 1143 and 84,506 patients received robotic-assisted and conventional THA, respectively. Following 1:1 propensity score-matching, there were 1143 patients in both cohorts for analysis (Fig. 1).

### Outcomes evaluated

Our primary outcomes were medical and orthopaedic complications occurring in varying timepoints: 30-day, 90-day, 1-year, and 5-year postoperative periods were assessed. Systemic complications included cardiac arrest, stroke, myocardial infarction (MI), acute kidney failure (AKI), surgical site infection (SSI), deep vein thrombosis (DVT), and pulmonary embolism (PE). Orthopaedic complications included revision, prosthetic dislocation, prosthetic joint infection (PJI), dislocation of hip/fracture of the femur, prosthetic pain, periprosthetic fracture, and aseptic loosening.

### Data analysis

All analyses were conducted within the TriNetX network. A 1:1 propensity score matching was conducted using a greedy nearest neighbor matching algorithm. The caliper distance was 0.1 pooled standard deviation of the logit of the propensity score. Patients were matched on the following demographics and comorbidities: age, race, ethnicity, sex, nicotine dependence, diabetes mellitus, primary hypertension, obesity, chronic lower respiratory diseases, kidney disease, anemia, and metabolic syndrome. Patient characteristics were considered well-matched if the *p *value was less than 0.05. Dichotomous outcomes were assessed using an odds ratio (OR) with a 95% confidence interval (CI). A *p *value less than 0.05 was considered significant.

### Patient characteristics

Characteristics of THA patients with at least 1-year follow-up and 5-year follow-up are provided in Tables 1 and 2, respectively. For 1-year follow-up robotic-assisted and conventional THA patients, there were differences in age (*p* =  < 0.001), race (*p* =  < 0.001), ethnicity (*p* =  < 0.001), nicotine dependence (*p* = 0.002), diabetes mellitus (*p* =  < 0.001), primary hypertension (*p* =  < 0.001), obesity (*p* =  < 0.001), chronic lower respiratory diseases (*p* = 0.001), kidney disease (*p* =  < 0.001), and anemia (*p* =  < 0.001) before propensity score matching. For 5-year follow-up robotic-assisted and conventional THA patients, there were differences in age (*p* =  < 0.001), race (*p* =  < 0.001), ethnicity (*p* =  < 0.001), diabetes mellitus (*p* = 0.001), primary hypertension (*p* =  < 0.001), kidney disease (*p* = 0.003), and anemia (*p* = 0.001) before propensity score matching. After propensity score matching, there were no differences between cohorts at 1-year and 5 years of follow-up (*p* =  > 0.05).

**Table 1:** Baseline patient demographics and comorbidities for robotic-assisted and conventional THA patients with minimum 1 year follow-up before and after propensity score matching

Variable: Avg. ± SD or n (%)	Before propensity score matching			After propensity score matching		
	Robotic-assisted (n = 2805)	Conventional (n = 162,265)	*p* value	Robotic-assisted (n = 2805)	Conventional (n = 2805)	*p* value
Demographics						
Age	63.3 ± 11.2	64.4 ± 12.4	**< 0.001**	63.3 ± 11.2	63.3 ± 11.2	0.802
White	2437 (86.9)	128,006 (78.9)	**< 0.001**	2437 (86.9)	2438 (86.9)	0.968
Not Hispanic or Latino	2382 (84.9)	113,419 (69.9)	**< 0.001**	2382 (84.9)	2376 (84.7)	0.823
Male	1394 (49.7)	69,010 (48.7)	0.291	1394 (49.7)	1390 (49.6)	0.915
Comorbidities						
Nicotine dependence	120 (4.3)	9174 (5.7)	0.002	120 (4.3)	110 (3.9)	0.501
Diabetes mellitus	176 (6.3)	15,072 (9.3)	**< 0.001**	176 (6.3)	178 (6.3)	0.913
Essential (primary) hypertension	667 (23.8)	52,511 (32.4)	**< 0.001**	667 (23.8)	667 (23.8)	1.000
Overweight, obesity, and other hyperalimentation	238 (8.5)	16,906 (10.4)	**0.001**	238 (8.5)	207 (7.4)	0.126
Chronic lower respiratory diseases	161 (5.7)	12,749 (7.9)	**< 0.001**	161 (5.7)	154 (5.5)	0.685
Acute kidney failure and chronic kidney disease	73 (2.6)	8805 (5.4)	**< 0.001**	73 (2.6)	71 (2.5)	0.866
Anemia	68 (2.4)	7436 (4.6)	**< 0.001**	68 (2.4)	65 (2.3)	0.792
Metabolic syndrome	11 (0.4)	881 (0.5)	0.280	11 (0.4)	10 (0.4)	0.827

**Table 2:** Baseline patient demographics and comorbidities for robotic-assisted and conventional THA patients with minimum 5 year follow-up before and after propensity score matching

Variable: Avg. ± SD or n (%)	Before propensity score matching			After propensity score matching		
	Robotic-assisted (n = 1143)	Conventional (n = 84,506)	*p *value	Robotic-assisted (n = 1143)	Conventional (n = 1143)	*p *value
Demographics						
Age	62.2 ± 12.6	63.5 ± 12.6	**< 0.001**	62.2 ± 11.1	62.3 ± 11.2	0.770
White	969 (84.8)	65,921 (78.0)	**< 0.001**	969 (84.8)	976 (85.4)	0.681
Not Hispanic or Latino	941 (82.3)	56,076 (66.4)	**< 0.001**	941 (82.3)	943 (82.5)	0.913
Male	595 (52.1)	41,796 (49.5)	0.081	595 (52.1)	595 (52.1)	1.000
Comorbidities						
Nicotine dependence	46 (4.0)	4461 (5.3)	0.059	46 (4.0)	42 (3.7)	0.664
Diabetes mellitus	65 (5.7)	7230 (8.6)	**0.001**	65 (5.7)	64 (5.6)	0.928
Essential (primary) hypertension	248 (21.7)	25,625 (30.3)	**< 0.001**	248 (21.7)	251 (22.0)	0.879
Overweight, obesity, and other hyperalimentation	85 (7.4)	7097 (8.4)	0.244	85 (7.4)	72 (6.3)	0.282
Chronic lower respiratory diseases	64 (5.6)	5968 (7.1)	0.055	64 (5.6)	55 (4.8)	0.397
Acute kidney failure and chronic kidney disease	31 (2.7)	3848 (4.6)	**0.003**	31 (2.7)	29 (2.5)	0.794
Anemia	26 (2.3)	3622 (4.3)	**0.001**	26 (2.3)	35 (3.1)	0.243
Metabolic syndrome	10 (0.9)	395 (0.5)	**0.046**	10 (0.9)	10 (0.9)	1.000

## Results

### Medical complications

After propensity-matching, the odds of developing any medical complications were not significantly different between RA-THA and C-THA at the 30-day, 90-day, and 1-year time points (Table 3). For patients with at least five years of follow-up, the risk of developing deep vein thrombosis was significantly lower in the RA-THA cohort (OR 0.5, 95% CI 0.3–0.9; *p* = 0.004) (Table 4). There were no other differences in the odds of developing medical complications at 5 years.

**Table 3:** Comparison of systemic complications in robotic-assisted and conventional THA at 1 year follow-up

Systemic complication	Robotic-assisted (n = 2805)	Conventional (n = 2805)	Odds ratio (95% CI)	*p* value
Cardiac arrest				
30 days	10 (0.4)	10 (0.4)	1.0 (0.4–2.4)	1.000
90 days	10 (0.4)	10 (0.4)	1.0 (0.4–2.4)	1.000
1 year	10 (0.4)	10 (0.4)	1.0 (0.4–2.4)	1.000
Stroke				
30 days	10 (0.4)	10 (0.4)	1.0 (0.4–2.4)	1.000
90 days	10 (0.4)	12 (0.4)	0.8 (0.4–2.0)	0.219
1 year	18 (0.6)	24 (0.9)	0.7 (0.4–1.4)	0.353
Myocardial infarction				
30 days	10 (0.4)	11 (0.4)	0.9 (0.4–2.1)	1.000
90 days	10 (0.4)	15 (0.5)	0.7 (0.3–1.5)	1.000
1 year	14 (0.5)	25 (0.9)	0.6 (0.3–1.0)	0.077
Acute kidney failure				
30 days	43 (1.5)	55 (2.0)	0.8 (0.5–1.2)	0.071
90 days	60 (2.1)	75 (2.7)	0.8 (0.6–1.1)	0.113
1 year	108 (3.9)	125 (4.5)	0.9 (0.7–1.1)	0.255
Surgical site infection				
30 days	15 (0.5)	15 (0.5)	1.0 (0.5–2.0)	0.601
90 days	28 (1.0)	29 (1.0)	1.0 (0.6–1.6)	0.520
1 year	37 (1.3)	47 (1.7)	0.8 (0.5–1.2)	0.272
Deep vein thrombosis				
30 days	20 (0.7)	23 (0.8)	0.9 (0.5–1.6)	0.554
90 days	32 (1.1)	34 (1.2)	0.9 (0.6–1.5)	0.724
1 year	42 (1.5)	49 (1.7)	0.9 (0.6–1.3)	0.459
Pulmonary embolism				
30 days	10 (0.5)	17 (0.6)	0.6 (0.3–1.3)	0.531
90 days	10 (0.4)	23 (0.8)	0.4 (0.2–0.9)	0.256
1 year	22 (0.8)	30 (1.1)	0.7 (0.4–1.3)	0.265

Odds ratios with *p* < 0.05 in bold

**Table 4:** Comparison of systemic complications in robotic-assisted and conventional THA at 5 years follow-up

Systemic complication	Robotic-assisted (n = 1143)	Conventional (n = 1143)	Odds ratio (95% CI)	*p *value
Cardiac arrest	10 (0.9)	10 (0.9)	1.0 (0.4–2.4)	1.000
Stroke	20 (1.7)	26 (2.3)	0.8 (0.4–1.4)	0.223
Myocardial infarction	19 (1.7)	21 (1.8)	0.9 (0.5–1.7)	0.431
Acute kidney failure	87 (7.6)	106 (9.3)	0.8 (0.6–1.1)	0.153
Surgical site infection	32 (2.8)	24 (2.1)	1.3 (0.8–2.3)	0.399
Deep vein thrombosis	21 (1.8)	41 (3.6)	**0.5 (0.3–0.9)**	**0.004**
Pulmonary embolism	11 (1.0)	18 (1.6)	0.6 (0.3–1.3)	0.430

Odds ratios with *p* < 0.05 in bold

### Orthopaedic complications

The risk of requiring a revision THA was significantly lower for the RA-THA cohort at the 90 days (OR 0.3, 95% CI 0.1–0.6, *p* =  < 0.001), 1 year (OR 0.3, 95% CI 0.2–0.5, *p* =  < 0.001) (Table 5), and 5 years (OR 0.4, 95% CI 0.2–0.8, *p* = 0.001) (Table 6). The RA-THA cohort had a lower risk of developing a prosthetic joint infection at 30 days (OR 0.5, 95% CI 0.2–0.9, *p* = 0.048) and 90 days (OR 0.5, 95% CI 0.3–0.9, *p* = 0.028). Additionally, the risk of prosthetic dislocation was lower in the RA-THA cohort at 90 days (OR 0.3, 95% CI 0.1–0.7, *p* = 0.002) and 1 year (OR 0.2, 95% CI 0.1–0.5, *p* =  < 0.001). The RA-THA cohort had a lower risk of developing a femur fracture or hip dislocation at 30 days (OR 0.5, 95% CI 0.3–0.6, *p* =  < 0.001), 90 days (OR 0.5, 95% CI 0.4–0.7, *p* =  < 0.001), 1 year (OR 0.5, 95% CI 0.4–0.7, *p* =  < 0.001), and 5 years (OR 0.6, 95% CI 0.4–0.9, *p* = 0.002). The odds of developing prosthetic pain were lower in the RA-THA cohort at 1 year (OR 0.4, 95% CI 0.2–0.8; *p* < 0.001) and 5 years (OR 0.4, 95% CI 0.2–0.8, *p* =  < 0.001).

**Table 5:** Comparison of orthopaedic complications in robotic-assisted and conventional THA at 1 year follow-up

Orthopaedic complication	Robotic-assisted (n = 2805)	Conventional (n = 2805)	Odds ratio (95% CI)	*p* value
Revision				
30 days	10 (0.4)	15 (0.5)	0.7 (0.3–1.5)	0.067
90 days	10 (0.4)	31 (1.1)	**0.3 (0.1–0.6)**	**< 0.001**
1 year	13 (0.5)	46 (1.6)	**0.3 (0.2–0.5)**	**< 0.001**
Prosthetic dislocation				
30 days	10 (0.4)	19 (0.7)	0.8 (0.3–1.9)	0.669
90 days	10 (0.4)	34 (1.2)	**0.3 (0.1–0.7)**	**0.002**
1 year	11 (0.4)	46 (1.6)	**0.2 (0.1–0.5)**	**< 0.001**
Prosthetic joint infection				
30 days	10 (0.4)	21 (0.7)	**0.5 (0.2–1.0)**	**0.048**
90 days	15 (0.5)	28 (1.0)	**0.5 (0.3–0.9)**	**0.028**
1 year	22 (0.8)	37 (1.3)	0.6 (0.3–1.0)	0.050
Dislocation of hip/fracture of femur				
30 days	39 (1.4)	93 (0.33)	**0.5 (0.3–0.6)**	**< 0.001**
90 days	64 (2.3)	124 (4.4)	**0.5 (0.4–0.7)**	**< 0.001**
1 year	82 (2.9)	151 (5.4)	**0.5 (0.4–0.7)**	**< 0.001**
Prosthetic pain				
30 days	10 (0.4)	10 (0.4)	1.0 (0.4–2.4)	0.335
90 days	10 (0.4)	10 (0.4)	1.0 (0.4–2.4)	0.335
1 year	14 (0.5)	31 (1.1)	**0.4 (0.2–0.8)**	**< 0.001**
Periprosthetic fracture				
30 days	31 (1.1)	17 (0.6)	1.8 (0.9–3.3)	0.123
90 days	36 (1.3)	27 (0.1)	1.4 (0.8–2.2)	0.626
1 year	44 (1.6)	35 (1.2)	1.3 (0.8–2.0)	0.308
Aseptic loosening				
30 days	10 (0.4)	10 (0.4)	1.0 (0.4–2.4)	1.000
90 days	10 (0.4)	10 (0.4)	1.0 (0.4–2.4)	1.000
1 year	10 (0.4)	15 (0.5)	0.7 (0.3–1.5)	0.316

Odds ratios with *p* < 0.05 in bold

**Table 6:** Comparison of orthopaedic complications in robotic-assisted and conventional THA at 5 year follow-up

Orthopaedic complication	Robotic-assisted (n = 1143)	Conventional (n = 1143)	Odds ratio (95% CI)	*p* value
Revision	10 (0.9)	26 (2.3)	**0.4 (0.2–0.8)**	**0.001**
Prosthetic dislocation	13 (1.1)	14 (1.2)	0.9 (0.4–2.0)	0.061
Prosthetic joint infection	22 (1.9)	29 (2.5)	0.8 (0.4–1.3)	0.878
Dislocation of hip/fracture of femur	46 (4.0)	76 (6.6)	**0.6 (0.4–0.9)**	**0.002**
Prosthetic pain	14 (0.12)	31 (2.7)	**0.4 (0.2–0.8)**	**< 0.001**
Periprosthetic fracture	25 (2.2)	29 (2.5)	0.9 (0.5–1.5)	0.575
Aseptic loosening	10 (0.9)	18 (1.6)	0.6 (0.3–1.2)	0.668

Odds ratios with *p* < 0.05 in bold

## Discussion

Although numerous studies have investigated RA-THA, most have small sample sizes, short-term outcomes, and consist of a surgeon’s first series of patients using a robotic platform, creating a need for a more widescale evaluation of RA-THA. Our analysis sought to investigate the outcomes of robotic-assisted THA in surgically routine cases examining medical and orthopaedic complications up to 5 years postoperatively by matching to conventional THA via a large, up-to-date national database. The analysis found comparable medical complication risks at all time points between RA-THA and C-THA Additionally, RA-THA showed reduced risks of revision, prosthetic joint infection, prosthetic dislocation, hip dislocation or femur fracture, and prosthetic pain. To our knowledge, this study is the first to examine the use of RA-THA in surgically routine cases and the results of this analysis encourage future utilization of RA-THA compared to C-THA for routine hip arthroplasty cases due to the significant reduction in complication rates.

Notably, a reduction in the incidence of revisions, prosthetic pain, and hip dislocations was found when undergoing RA-THA compared to C-THA at the 1- and 5-year postoperative time points. These findings contrast Sweet et al. [24], which reports no difference in postoperative dislocations, complications, or revision rates. Likewise, a similar meta-analysis also reported no difference in complication rates between RA-THA and C-THA overall as well as between RA-THA with the MAKO system versus C-THA [25]. However, due to each study’s small sample size, the studies included in both meta-analyses had very few complications or revisions in total, lacking power to detect any difference. As dislocation, instability, and pain are major issues that contribute to the necessity of revision THA [26], the decreased incidence of revisions found in this study with RA-THA may be partially explained by the well-documented superior accuracy of RA-THA over C-THA for cup placement [27–30]. Furthermore, RA-THA has been shown to improve positioning in patients with a large body habitus [31–33].

While there is a decrease in orthopaedic complications, patients may or may not perceive a benefit. In a prospective cohort study with a minimum of three years’ follow-up, Fontalis et al. [34] identified that RA-THA had improvement in accuracy of acetabular component placement; however, this did not translate to statistically improvement in Oxford hip score or University of California at Los Angeles scores. Furthermore, a prospective study of 60 patients divided into RA-THA and C-THA found no difference in patient reported outcome measures (PROM) at three months postoperatively [35]. Contrary to those studies, a retrospective review of 9892 primary THA cases identified that robotic cases had the greatest improvement in the hip dysfunction and osteoarthritis outcome score, joint replacement, and the patient-reported outcome measurement information system pain interference scores at both six weeks and three months postoperatively and were more likely to have same-day discharge [36]. Further research ought to be done to identify if the reduction in complication translates to perceived benefit by the patient.

Medical complications between RA-THA and C-THA rates at all intervals assessed in this study were similar. In particular, there were comparable rates of superficial SSIs between the two matched cohorts, even though RA-THA is associated with increased operative time [37], and longer operative times have been associated with increased incidence of superficial SSIs [38]. This is supported by Ng et al. [39]. Therefore, in addition to operative time, surgeon ability, patient comorbidities, and case complexity should be considered when assessing the safety of RA-THA. Given the comparably low rate of complications reported for both RA-THA and C-THA, analyzing other metrics may provide more insight into which THA technique should be regarded as superior. For instance, prior meta-analyses have shown that patients undergoing RA-THA have better radiographic outcomes, including higher rates of cup placement within the Lewinnek and Callanan safe zones [29, 40]. Cup malposition has been associated with increased odds of hip dislocation, worse biomechanics, and poorer functional outcomes [29, 41, 42], so evaluating RA-THA against C-THA may be best conducted within these domains.

### Limitations

This study has some limitations. First, the study utilized a database reliant on data retrieved from electronic health records across various systems, leading to potential coding discrepancies and underreporting complications. The risk for incorrect diagnosis and clinical event coding exists as a result, and ICD codes occasionally may not be sensitive enough to identify all relevant adverse events. However, despite this limitation, we found significant increases in the odds of medical and orthopaedic complications among C-THA patients compared to RA-THA patients. Second, variations in surgical materials, techniques, and postoperative care protocols among health systems as well as surgical skills could influence findings. Third, we were unable to segregate cases based on implant type, robotic platform utilized, surgical approach, or surgeon experience. Lastly, our study was limited by its retrospective nature and there may have been additional variables or confounding effects that were not included in the database. These complications decrease the likelihood that the complications can solely be attributed to RA-THA compared to C-THA. It is unlikely the strong decrease in complications with RA-THA is caused by factors such as skill as the learning curve associated with RA-THA is attributed to an increased operative time and increased error rate in acetabular component placement [39].

## Conclusion

This study offers insights into RA-THA’s performance in cases of reduced surgical complexity. Our analysis found comparable medical and orthopaedic complication risks at 30 days, 90 days, 1 year, and 5 years between RA-THA and C-THA. However, RA-THA demonstrated decreased odds of revisions, prosthetic pain, and hip dislocations at 1- and 5-year timepoints. Future studies with large sample sizes and long-term follow-up are needed to understand the patient-reported outcomes and functional outcomes of RA-THA for cases with reduced surgical complexity to better understand the advantages that RA-THA may offer over C-THA.
